# Wildlife Trade and Law Enforcement: A Proposal for a Remodeling of CITES Incorporating Species Justice, Ecojustice, and Environmental Justice

**DOI:** 10.1177/0306624X221099492

**Published:** 2022-05-19

**Authors:** Ragnhild Sollund

**Affiliations:** 1 University of Oslo, Norway

**Keywords:** CITES, wildlife trade, justice, green criminology, animal abuse

## Abstract

Wildlife trade is an increasing problem worldwide, whether legal or illegal. It causes species extinction, connects to organized crime and contributes to social unrest. Wildlife trade is regulated through the Convention on International Trade in Endangered Species of Wild Fauna and Flora (CITES), a convention that includes most of the countries in the world. Even though wildlife trade is not necessarily breach of any law, wildlife trade still constitutes severe breaches of species justice, ecojustice, environmental justice, and animal rights. By employing these perspectives in the study of wildlife trade, the harms nonhuman animals suffer as victims of this trade receives a broader concern than that encompassed through conventional criminology. This article addresses nonhuman animal victimization through a theoretical lens that includes the justice perspective found in green criminology, and Nussbaum’s concept of dignified existence. Empirically the article is based on an ongoing research project: Criminal Justice, Wildlife Conservation, and Animal rights in the Anthropocene (CRIMEANTHROP). The article starts with an introduction, followed by theoretical outlining and a presentation of empirical findings. These findings are discussed using the theoretical perspectives mentioned above. The concluding discussion suggests a radical shift in the function of CITES, from trade to conditional aid.

## Introduction

Wildlife trade harms and kills billions of animals^1^ yearly. The phenomenon is multifarious. It includes the hunting and fishing of animals for food (e.g., Kurpiers et al., 2016); trophy hunting (Sollund & Runhovde, 2020; Wall & McClanahan, 2015); and the exploitation of animals, such as of pangolins, moon bears, and tigers, who are encaged and whose body parts and derived products are used in Traditional Chinese Medicine (TCM) (e.g., van Uhm & Wong, 2019). Animals and animal products are also traded for other purposes, much of which/whom is trafficked in luggage to Europe (Sollund & Maher, 2015; van Uhm, 2016) or to the US (Petrossian et al., 2016). Elephants are killed for their ivory (Underwood et al., 2013) or trafficked to circuses and zoological gardens (McKenzie & Swails, 2019); birds and their eggs are taken to become part of collections (Sollund, 2019). Live animals are trafficked broadly to be used as domestic “pets” (Lavorgna, 2014; Sollund, 2019; van Uhm, 2016; Wyatt, 2021b).

The national and international trade in wild caught animals is widespread. Most of these animals suffer from this, whether reptiles (Warwick, 2014), mammals or birds. Reptiles are hunted and skinned, or locally bred and skinned, to become fashionable purses, belts and shoes. They are often kept in cramped and filthy conditions (Marshall et al., 2020). The captive breeding of crocodiles is an important source of income for, for example, Colombia (Sollund, 2019); however, a large number of wild caught crocodiles and caimans are trafficked for the same purpose (Sinovas et al., 2017).

Wildlife trade in general is not forbidden, however many species are protected from hunting in countries’ national legislation. Wildlife also receive partial protection in the Convention on International Trade in Endangered Species of Wild Fauna and Flora (CITES). CITES is an international trade convention *regulating* wildlife trade that was established in order to prevent the extinction of species when, in the 1960s, worries arose that the global wildlife trade was increasing to such an extent that countries in the South would lose the resources wildlife (plants and animals) were regarded to constitute for many nations (CITES n.d.). CITES currently has 183 parties. CITES does not protect animals from trade *before* their species is threatened with extinction. CITES also has little concern for the welfare of *individual* animals who are victims of trade (Goyes & Sollund, 2016; Mulà Arribas, 2015; Sollund, 2019; Wyatt et al., 2021), and partial protection is awarded only to those species that are listed on the CITES appendices. Currently 5,950 species of animals and 32,800 species of plants have some protection in CITES, but far more species never reach any appendix. This is only one of the weaknesses of CITES (Reeve, 2014, Goyes & Sollund, 2016, Sollund, 2019, Wyatt, 2021a, 2021b).

Species are grouped in the appendices according to how threatened they are by international trade. Appendix I includes species threatened with extinction. Trade in these species is permitted only in exceptional circumstances. Appendix II includes species not yet threatened with extinction, but in which trade is controlled in order to avoid exploitation incompatible with species survival. Appendix III species are those that are protected in at least one country, which has asked other CITES Parties for assistance in controlling the trade. Parties to the convention must implement the convention through national legislation and through the establishment of a management authority. In Norway, currently, CITES is implemented through the Nature Diversity Act under the management authority of the Norwegian Environment Agency (henceforth NEA).

CITES is an anthropocentric trade convention objectifying animals (Goyes & Sollund, 2016; Sollund, 2019), which is out of touch with what is known about the cognitive abilities of animals (e.g., Mertz et al., 2019; Pepperberg, 2009) and their needs. This constitutes one main weakness of the convention. It is also a general problem concerning the implementation of the convention that many parties fail to fulfill their obligations, such as submitting the annual and biannual trade reports to the secretariat (Wyatt, 2021a, 2021b). What is actually traded legally remains largely in the shadows. Because most species are not subject to any regulation, many species are going extinct without ever being listed or even discovered (e.g., Frank & Wilcove, 2019). CITES can thereby create an illusion that wildlife trade is subject to control and enforcement that are actually lacking.

In this article, I will discuss the lack of moral and species justice existing in the enforcement of the illegal wildlife trade, while not disregarding the harms that are inherent in the legal trade. Two issues are particularly salient in Norway: the partial lifting of the ban against exotic reptiles, and the state policy of killing confiscated victims of illegal wildlife trade as an enforcement measure. These two issues lead to a discussion containing a proposal for a plan about a remodeling of CITES. First, I will present the theoretical framework and the methodology for the research I base this article on, before presenting findings and finally discussing these in view of the theoretical perspectives.

## Theoretical Framework

I situate this article within the field of green criminology. This is a subfield of critical criminology that acknowledges that what is defined as crime is socially constructed and embedded. Harms that are not criminalized can be as harmful as acts that are criminalized, and because what is criminalized varies in different times and within different social communities, green criminology scholars study *harms* as much as *crimes* (e.g., Brisman & South, 2014; Lynch & Stretesky, 2016; Sollund, 2015; White, 2013). The concept of harm is particularly relevant in the context of wildlife trade because everything an animal is exposed to as part of this trade is harmful (Sollund, 2011; Wyatt et al., 2021), yet most of these harms take place within the limits of the law. For example, every year thousands of parrots are legally abducted and sent from Latin America to countries such as Russia and Singapore to be “pets” in private households (Sinovas et al., 2017). This is extremely abusive because parrots are highly intelligent beings (Pepperberg, 2009) who are not adapted to live in a cage in an apartment, nor to be disrupted from their flock structure or to be trafficked long distance as cargo.

Central in the concept and study of harm envisioned as zemiology, introduced to critical criminology with Hillyard and Tombs’ work in 2004, are the crimes of the powerful. This usually refers to human social structures, where states and large corporations cause harm in and through capitalist systems (e.g., Stretesky et al., 2013; Tombs & Whyte, 2020). Those who are most victimized are, however, non-human species. Speciesism is oppression based in the fact that nonhuman animals are perceived as having less value than humans (Singer, 1995). In addition, there are hierarchical differences among nonhuman species. Speciesism is a system of belief, practice and ideology rooted in philosophy and religion and one of the most fundamental features of all human societies (e.g., Nibert, 2002, 2013; R. A. Sollund, 2016). Wildlife trade and other forms of animal abuse can therefore be seen as structural violence (Galtung, 1990).

Features of speciesism that legitimate animal oppression are *difference*: nonhuman animals are physically different from the human animal; *distance*: nonhuman animals are often physically and socially apart from humans; and *denial*: “animals do not suffer like humans” (Sollund, 2008).

The animal abuse and structural and systemic violence of wildlife trade can also be perceived as a state-corporate crime. Tombs and Whyte (2020) use pollution and the climate crisis as examples of state corporate crime produced by the logic that capital must be relatively free to reproduce itself, no matter the cost. For example, causing death through exposure to pollutants is an “inevitable,” normal effect of legal productive activity, and one that is largely permitted by systems of regulation. Likewise, generally wildlife trade is *regulated*, not *criminalized*, and the root motivation for the large majority of trade is economic gain, albeit at different levels. For example, Zimbabwe as a state benefits largely from the trophy hunting industry, while the various states that are parties to CITES also facilitate the harms of wildlife trade in order to allow individuals and human communities to benefit economically.

Another central theoretical perspective in green criminology is that of animal rights and species justice. A bearer of rights must be recognized for his/her inherent value, not by the value s/he provides for a human; whether the bearer is a human or a nonhuman animal is or should be irrelevant because most animals possess the ability to suffer, feel joy, sorrow, and fear and are subjects in their own lives (Benton, 1998; Regan, 2004). Animals who can suffer equally to humans should be accorded the same concern (Singer, 1995). Animal rights should entail that a human has no right to inflict harm upon an animal for his/her own benefit, or to treat an animal as property (Francione, 2008). This is a radical point of view and the subject of much debate, for it raises questions, such as: How far should rights reach? Who would be responsible for protecting these rights? What should they consist of? Which species should have rights? There is for example The United Nations Educational, Scientific and Cultural Organization [UNESCO] declaration of animal rights (UNESCO, 1978).^2^ It establishes in Article 1 that “All animals have equal rights to exist within the context of biological equilibrium. This equality of rights does not overshadow the diversity of species and of individuals,” and in Article 2 that “All animal life has the right to be respected.” This declaration was produced only a few years after CITES was implemented and the two are contradictory, although there are moderations to the rights animals are entitled to in other articles of the UNESCO declaration, introducing concepts like “unnecessary” and “unjustifiable killing,” which imply that some killing and some harm are necessary and justifiable. Article 3 offers particular protection to wildlife: “Wild animals have the right to live and to reproduce in freedom in their own natural environment. 2°- The prolonged deprivation of the freedom of wild animals, hunting and fishing practised as a pastime, as well as any use of wild animals for reasons that are not vital, are contrary to this fundamental right.” Article 4 is also very important for wildlife: “Any act compromising the survival of a wild species and any decision leading to such an act are tantamount to genocide, that is to say, a crime against the species. 2°- The massacre of wild animals, and the pollution and destruction of biotopes are acts of genocide.” Genocide is also ecoviolence (Stoett & Omrow, 2020) that reaches beyond the suffering of each individual who is victimized by humans in the wildlife trade.

All killing, hunting and fishing violate animal rights. Clearly many entities and persons are acting under the regulatory body of CITES, which thereby is fundamental for the billion breaches of animal rights in this regard, because killing, capturing and very often abusing and exploiting animals is the essence of CITES and the basis on which it relies. To the degree that CITES contributes to species extinction (Goyes & Sollund, 2016), CITES definitely deserves to be linked to organized, state corporate crime.

Justice is central to rights. White (2013) elaborates on perspectives of justice that have become central in green criminology and that are highly relevant concerning wildlife trade. Ecological citizenship and ecological justice acknowledge ecosystems that should be preserved for their own sake via the notion of the rights of the environment. Animal rights and species justice are constructed in relation to the place of nonhuman animals within environments and their intrinsic right to not suffer abuse, whether this be one-on-one harm, institutionalized harm, or harm arising from human actions that affect climates and environments on a global scale.

By “harm,” I imply ill treatment that impairs physical and/or mental health, or/and an individual’s physical, mental, or emotional development and/or social behavior, including the opportunity to act out physical, emotional, and social species-specific needs. Harm can be caused by direct or indirect actions or omissions and neglect. Any such harm is a breach of species justice and animal rights (see below) and impairs the animals’ capabilities and thus their ability to act in the ways that are natural for them, to act with agency and dignity.

These perspectives acknowledge the role wildlife play in their natural environment in preserving ecosystems, and their rights to remain within them as these environments’ rightful citizens (Donaldson & Kymlicka, 2011) rather than being killed and/or forcefully abducted, and that they have the rights not to be victims of harm and theriocide (Beirne, 2014). To acknowledge that animals are victims of theriocide and abuse in wildlife trade implies including them in our moral circle (Donovan, 1996) and recognizing them as victims, in line with the critical victimology found in green criminology (Beirne, 2014; R. Sollund, 2016; White, 2013, 2018). Several philosophical perspectives can be incorporated into green criminology because of their common ground.

Nussbaum (2006a, 2006b) adopts a “capability” approach when she discusses justice. It begins with asking; “What are people actually able to do and to be?” She maintains that each human being should be entitled to a decent level of opportunity in areas of particular centrality, such as life, health, bodily integrity, social belonging, and their ability to reason. She then extends this to non-human animals because a capabilities approach can recognize a wide range of types of animal dignity, and what animals need in order to flourish. This includes for example (also), the ability to move, bodily integrity and to avoid pain, as well as affection, health, and a community. Nussbaum connects the satisfaction of such needs with dignity. The basic moral intuition behind this approach concerns the dignity of a form of life that possesses both deep needs and abilities, and the plurality of activities and needs must be taken into account for all life (Nussbaum, 2006a, 2006b). To deny an animal respect is an issue of justice when s/he does not have the opportunity to unfold her (valuable) power, to flourish in her own way, and to lead a life with dignity. Denying billions of animals, for example, the opportunity to move around, enjoy the air and exchange affection with other members of their kind is a waste and a tragedy, and deeply undignified (Nussbaum, 2006b). A panther stereotypically pacing to and forth in a 2 m × 3 m cage in a zoo, like I once witnessed in Prague, is an example of a wild animal prevented from out acting agency, forced to live an undignified existence exposed to the curious human gaze.

Nussbaum’s theory of justice and her “capability” approach differs from Tom Regan’s perspective of animals as “moral patients.” According to Regan, moral patients lack the prerequisites enabling them to control their behavior in ways that would make them morally accountable for what they do. This means that animals do not have moral, something which is refuted for example, by de Waal’s studies on bonobos (De Waal, 1996). Even when a moral patient causes significant harm to another, the moral patient, according to Regan, has not done wrong because only moral agents can do what is wrong. Regan perceives human infants, young children, and the mentally infirm as human moral patients. When Regan positions animals as moral patients alongside children, he implies that neither are able to make moral choices—something many people who have closely observed children and animals would dispute. To be a patient also have connotations to passivity and disability, in contrast to moral agents, who act.

Nussbaum has a focus on agency and what animals can do, not what they cannot do. I could mention numerous examples that exceedingly surprise researchers concerning the abilities and skills of animals documenting agency. I will settle with one: A group of animal ecologists had equipped Australian magpies with electronic devices in order to track them. The goal of the experiment was to track the movement and social dynamics of these highly intelligent birds, and to test the new, durable and reusable devices. However, the birds surprised the researchers in that they quickly helped each other in removing the devices, thus displaying both altruism and cooperation, what the researchers called cooperative rescue behavior (Potvin, 2022). It illustrates also that these birds and many other species are social beings who suffer in captivity, as they are unable to relate socially and with skills to their fellow beings.

Regan clearly states that, many animals have abilities that by far exceed the abilities of babies, people with dementia, or otherwise disabled, however, he still argues that animals can be seen as moral *patients*. In my view, animals are not patients except when they are new born, and even then, many animal species far outreach the capacities of new born human babies. They *become* moral patients when/if they are put in a disabling position, through which they become dependent on human care. When captured and encaged, animals are forcefully transformed from being active agents to moral patients. This is to large degree what captivity of animals consists of and happens frequently in wildlife trade. As part of the trade, animals who are kept alive are put into a helpless situation where they cannot use their skills and abilities. They are deprived of all the facets a life as a free individual offers—be it dangerous at times, even lethal, but also full of joy, victories, love, and peace. Thus is an act of tremendous injustice. It means that an individual is deprived of reaching her potential—a breach of the fundamental rights mentioned in the UNESCO declaration of animal rights.

## The Partial Lifting of the Ban Against Exotic Reptiles in Norway

On August 15, 2017, an important policy change concerning wildlife trade took place in Norway that connects directly to the harms and injustices implicit in the trade, when a former ban on reptile trafficking in Norway was partly lifted and a positive list introduced. A positive lists involve the itemization of animal types that are permitted for trading and keeping, with all nonlisted animals essentially being barred (Warwick & Steedman, 2021). For many years, Norway and Iceland had been the only countries that prohibited the keeping of exotic reptiles. Of the 19 reptile species that were permitted in trade and to be kept as household animals, 15 are either CITES-listed or listed as vulnerable on the International Union for Conservation of Nature (IUCN) list. These also involve species that are already heavily trafficked, such as boa constrictors, ball pythons, the giant Madagascar day gecko, and the Central bearded dragon.

**Table.** Snakes permitted as household animals in Norway.

Name	Norwegian Name	Species	Status
Green tree python	grønn trepyton	*Morelia viridis*	CITES II
Garden boa	hagetreboa	*Corallus hortulanus*	IUCN listed
Boa constrictor	kongeboa	*Boa constrictor*	CITES II
Ball python	kongepyton	*Python regius*	CITES II
King snake	kongesnok	*Lampropeltis getula*	IUCN red list
Corn snake	kornsnok	*Pantherophis guttatus*	IUCN red list
Milk snake	melkesnok	*Lampropeltis triangulum*	IUCN least concern
Rainbow boa	regnbueboa	*Epicrates cenchria*	CITES II
Carpet Python	teppepyton	*Morelia spilota*	CITES II

**Table.** Lizards permitted as household animals in Norway.

Name	Norwegian Name	Species	Status
Spiny-tailed monitor	dvergvaran	*Varanus acanthurus*	CITES II
Crested gecko	kranset gekko	*Correlophus ciliatus, Tidligere Rhacodactylus ciliatus*	IUCN red list
Leopard gecko		*Eublepharis macularius*	IUCN least concern
Ocelleated lizard	perlefirfisle	*Lacerta lepida*	IUCN red list
Eye-dabbed lizard	pigghaleagam	*Uromastyx ocellata*	CITES II
Central bearded dragon	skjeggagam	*Pogona vitticeps*	IUCN least concern
Giant Madagascar day gecko	stor daggekko	*Phelsuma madagascariensis*	CITES II

**Table.** Tortoises permitted as household animals in Norway.

Name	Norwegian Name	Species	Status
Greek land tortoise	gresk landskilpadde	*Testudo hermanni*	CITES II
	kinesisk trekjølskilpadde	*Chinemys reevesi*	CITES III
	rødfotet skogskilpadde	*Chelonoidis carbonarius*, previously *Geochelone carbonaria*	CITES II

The arguments of the Food Safety Authority (FSA), which is the entity responsible for animal welfare and food safety in Norway, were that for many years and despite the ban, an estimated number of 100,000 reptiles were already in private households. It was also difficult to ensure welfare for these because owners might have been reluctant to bring their animals to veterinarian care for fear of penalties. There are still restrictions. Wild animals that have been caught/captured are prohibited from trade and owners must provide a document from the salesperson verifying that the animal has been bred in captivity. In addition, CITES documents must be in order if the species is CITES listed. According to interview data,^3^ these documents provide little certainty that animals are not wild caught because all it takes is for a seller, such as someone at the infamous reptile fair in Hamm, Germany, which is known for commercializing wild caught reptiles (Sollund, 2019), to attest that he has bred the animal himself. In this way, wild caught animals may be laundered into traffic, pass as legal and threaten species conservation (Sollund, 2019).

Another problem with the legalization of reptiles in Norway is that they have now become easily accessible and more people, who would refrain from buying these animals before due to the ban, may now buy them on a whim—especially because they are sold on websites, such as Finn.no. For example, at the time of writing, December 1, 2021, there were 17 advertisements for reptiles, along with others advertising CITES-listed parrots and other animals used as companions. Typically, and like many other species that are commercialized as “pets,” such as parrots who may be challenging to keep due to noise and destruction of inventory, reptiles are often second-hand animals, often having had multiple owners. The internet has increased the availability of exotic animals (International Fund for Animal Welfare, 2008; Lavorgna, 2014), whether the species are legal or illegal in trade. The internet also increases the attraction exotic “pets” have for consumers though videos posted on social media, such as Facebook and YouTube (Nekaris et al., 2013). Collectors may claim their activity to be “an expression of a desire to reconnect with the natural world, and that ownership—especially of endangered species—is not a form of exploitation but an act of ‘conservation’ and an important way of keeping such animals alive on Earth” (Brisman & South, 2020: 926). The commercialization of animals that are not adapted to living in human environments increase the danger of animal abuse and species injustice (Sollund, 2011, 2019; Wyatt et al., 2021). One example of the consequences of the opening of reptile trade in Norway came across in interviews.

A border veterinarian at Oslo airport found that there is a big problem with the import of live reptiles, and that this had very little priority among law enforcement agencies. She said:

“There is a pattern concerning the importers who are often individuals who are registered with a single person enterprise [enkeltmannsforetak], that have bad routines for keeping and transporting the reptiles. One case among many concerned reptiles who were sent from the Netherlands. It was winter and cold, and when they arrived, all 22 reptiles were dead. They were transported in an ordinary paperboard box. According to the transporter, they were placed outside awaiting transport and froze to death. The person behind this import, however, was a private individual who imported them. And there should ring some bells, for what should one person want with 22 reptiles?”

The veterinarian implied that the private individual was importing reptiles in order to sell them online and she found the now legal reptile trade abusive, as well as lethal. Even if the reptiles had survived and been sold to random people through the internet, the chance that they would have had a good life is minimal. According to Warwick (2014), the reptile trade raises concerns about breach of animal welfare, such as associated with handling, storage, transportation, intensive captive breeding, captivity stress, injury, disease, and high premature mortality. Reptiles’ needs may be hard to accommodate and many reptile keepers are associated with crimes that are incompatible with animal welfare, such as drug crimes and violence (Sollund, forthcoming).

Anyone who wishes to set up a breeding facility or start as a vendor of reptiles in order to make a business can easily do so, with very limited control from the FSA.

Another apparent breach of animal rights and species justice is the Norwegian policy of killing animals who are seized in traffic on Norwegian borders. This has also been the case with almost all reptiles who were seized, such as when the police found them when searching an offender’s house for other reasons (Sollund, forthcoming). This systematic killing was also a reason why the FSA wanted a lift of the ban. One interviewee stated: “Many in the FSA find it unpleasant to euthanize perhaps well kept, healthy animals just because they belong to an illegal species.”

It is still illegal to buy and sell many species, however, and for the individual animals, the enforcement measure that most frequently is used in combination with the administrative sanction of a fine, is theriocide of the trafficking victims (Sollund, 2021). This occurs regardless of whether the species is listed on Appendix I of CITES as endangered.^4^ A border veterinarian told me of two such incidents and how she experienced having to carry out this policy:

First, one man arrived with three parrots (*Eclectus roratus*) listed in Appendix II, without CITES documentation. The veterinarians wanted to rehome them at a zoological garden, but because the NEA is the CITES authority, the decision was theirs to make. The rules of the FSA are that it is permitted to import up to three birds provided they are accompanied with the CITES documents. When the veterinarians asked about the rehoming options, the NEA advisor said that, “We have as a policy when the animals have arrived to Norway without papers, that only one thing matters, which is to ‘euthanize’ them.” So, the veterinarians had to kill them.

Then, shortly thereafter, another man arrived with three African grey parrots to Oslo airport: The veterinarian elaborates:

“And shortly after, a new idiot—to put it bluntly—arrived, with three African Grey Parrot (*Psittacus erithacus*) babies. My colleague had the responsibility for hand feeding them. And they are so smart, like five-year-old kids. And I, I get . . . I have euthanized many animals, but I cried when I euthanized these birds. Because I thought it was hell euthanizing them. They understood. It is completely different with a dog, they don’t understand anything. But these, they understood, as my colleague and I entered, that this was big shit. So, we had to euthanize three more parrots. And what kind of handling of endangered species is that? I thought it was terrible. (. . .) I think it is a totally wrong way of enforcement. It goes against all I stand for. To take these birds out, it was so different from euthanizing other animals, I thought; they understand. [But] we do not want to work as the NEA’s executioners of endangered species. And it really isn’t our role either. It is not ok that the NEA make decisions of euthanizing without seeking other alternatives.” (Sollund, 2021).

To kill animals of an endangered species is a crime. This is established in the Norwegian Nature Diversity Act, through which CITES is implemented in Norway and enforced. Despite this, and despite Norway’s obligations to protect endangered species, animal victims of trafficking who are endangered are routinely killed by veterinarians who are forced to commit these acts of theriocide that contravene their own convictions and professional ethics.

## No Rights, No Justice

When the NEA orders these killings to take place, it is a representative of a regulatory regime that allows harms and crimes to be perpetrated. This should lead us to understand such harm or crime as “state-corporate” in character (Tombs & Whyte, 2020, p. 21). This resonates with Norway’s alliance with CITES, which lays the ground for the trafficking of animal corpses of endangered species as trophies and the trade in exotic CITES listed species more generally. This, and the ways in which CITES is enforced, should also be seen from the perspective of Norway’s lack of fulfilment to the country’s obligations to the Bern Convention.^5^ This can be perceived as state-organized crime caused by ideological inertia (Sollund & Goyes, 2021). Inherent in this inertia is the failure of acknowledging nonhuman animals as bearer of rights and entitled to justice (Nussbaum, 2006), due to their being subjects in a life as sentient beings (Regan, 2004), with interests in pursuing their life as they were meant to, unharmed by humans.

The animals who are seized have an interest in life, they have only one. The African Grey babies were probably bred in Spain from where they were trafficked. Even though they would obviously be better off living a life in their natural habitats rather than in in a human environment (such as someone’s apartment), their reaction, as described by the veterinarians, indicates that they wanted to live and therefore killing them was not an act of mercy, which the term *euthanasia* may imply, but a crime. They were killed to make a point and to get rid of a problem, and such killing is also used to deter individual offenders from reoffending (Sollund, 2021). They were deprived of a potential lifespan of 180 years, 60 each, which underlines the speciesism that is the basis for the enforcement of CITES in Norway, as well as the speciesism that has shaped the convention itself.

The central eco-crime unit of the Norwegian police [Økokrim]^6^ acknowledged in an interview that this kind of enforcement is problematic:

“It is certainly a dilemma that there is a regulation intended to protect species from trade, and that there is no apparatus to receive the species that are seized or revealed, or to handle them in other ways. And the trade is intercepted precisely in order to protect the species.”

The paradox in this way of enforcement that underlines the lack of recognition of individual animal rights is also apparent in the wildlife trade of exotic “pets,” more generally. It entails an objectification and encaging of individuals who have often been free born or who are not adapted to a life in a cage—just like humans are not adapted to a life in a cell (e.g., Arrigo et al., 2011; Christie, 2007). An example of the process by which victims of wildlife trafficking are turned from active agents to moral patients—incapacitated and helpless, but yet regarded as, and perhaps *because* of it receiving a form of “care”—comes through very well in a study about the bird trade in Mexico (Roldán-Clarà et al., 2017).

The bird abductors and traffickers are presented as skilled and maintaining traditions by the authors, but the methods described to catch the endangered birds and the ways in which they are “acclimatized” seem torturous. For example, no attention is paid to the harms done to the birds by breaking up their families and flock structures. Traffickers state that the birds must be accustomed to a new diet, which means they are forced to eat other food than that they are used to and which is right for them. This means that the birds are fed the *wrong* food, causing malnutrition and disease, and ultimately premature death.

In order to “tame” the birds, they are isolated. During such procedures, the birds become depressed from stress—a natural consequence of being captured and deprived of their usual food, freedom, flock, and natural habitat. Traffickers refer to this as the birds being “spoiled and sad” (*se chiquean, se ponen tristes*). Birds who refuse to eat the new food are force fed. The bird abductors respond to the birds’ natural reactions, such as depression and refusing to eat, by feeding them antibiotics and covering their cages with a cloth (Roldán-Clarà et al., 2017).

In essence, any free born animal who is captivated and maintained this way is put in a helpless situation. This is defined as a crime in the Norwegian Animal Welfare Legislation’s §14,b.^7^ They are deprived of the ability to pursue their social, psychological and physical needs, which is an obvious breach of species justice (Nussbaum, 2006; Sollund, 2019; White, 2013). When they are abducted and encaged, they are indeed turned into helpless moral patients, when they should be entitled to rights (Regan, 2004). In Norway, as in most other countries that are partners to CITES, they are objectified as having no interests, nor rights at all.

## From Trade to Aid?

Rather than allowing for more animals to be traded as commodities, the authorities should promote new forms of relations with nature and non-human animals that are not based on their being disposable property of humans; they should be treated as beings with their own dignity (Nussbaum, 2006) and intrinsic value (Regan, 2004). As stated by Benton (1998):

“The aim would be to create harmonious, benevolent and co-operative spirals, against destructive, competitive and antagonistic ones, through the establishment of new dominant patterns of interaction, experiences and spontaneous sentiments” (Benton, 1998, p. 171).

For example, wildlife could be admired in their natural surroundings if this can be done without humans disturbing them or without other destructive harm to the environment (see, e.g., Clifton & Benson, 2006; Wight, 1993), rather than them being held captive in urban, human environments, as human property, entertainment and adornment.

As said, wildlife trade is regulated through CITES. Unfortunately, CITES is, today, malfunctioning, even according to its own principles (Wyatt, 2021a, 2021b). Due to the harms involved in wildlife trade and the ways in which it is organized through CITES and implemented at national levels, I suggest defining the legal wildlife trade as a *transnational, global*, *organized*, *state corporate crime*. I thereby imply that animals should have rights *not* to be exploited as household animals, killed for their flesh or skin, teeth or whiskers, tusks, horns, or used for entertainment in zoos, circuses, and aqua parks.

Through the organization of wildlife trade, state institutions (like the management authorities of CITES’ parties, such as the Norwegian Environment Agency), directly and indirectly cooperate with states and private corporations (e.g., trophy hunting companies in South Africa and Zimbabwe; zoological gardens), in killing and trading animal victims, including endangered species. The idea behind CITES is that the harms of wildlife trade shall continue as an eternal chain through which continuously new individuals can be abducted, killed or in other ways exploited in a “sustainable” way for human benefit.

This situation does not have to endure. A key element in the creation of new human-animal-natural environment relations (Benton, 1998) is to address the (mal)functioning of CITES, which is rooted in species injustice and exploitation. CITES could become an instrument promoting environmental justice, ecojustice, and species justice (Sollund, 2019; White, 2013), if it were to be transformed from a *trade* convention to an *aid* convention. Trade, not aid was a mantra that came into fashion in the late-1980s/early 1990s, with the idea that sustainable trade involving “developing” countries should replace the nonprofitable humanitarian aid regime. With the Brundtland report on sustainability^8^ and with support from the International Monetary Fund (IMF) and World bank, the scampi industry, for example, exploded, causing environmental harm and breaches of human rights (O’Brien, 2008). Although trade rather than aid may seem like a better choice for countries that seek economic independence, as shown by O’Brien (2008), the consequences were dire. CITES preceded the Brundtland report, but sustainable trade is still a clue in the anthropocentric ideology of CITES. In this logic, one individual can easily be disposed of and replaced by another, the intrinsic value of all individuals is not recognized.

As mentioned above, the rationale behind CITES is that wildlife are *resources* whose trade shall be regulated so that species can be conserved primarily for human benefit. The rationale of the convention could be reformulated to promote species’ conservation and the protection of individuals’ and species’ rights. This is essential in the preservation of non-human animal species and ecosystems, and of course for all sentient beings. Environmental justice, seen as a prolongation of human rights (e.g., White, 2013), could be promoted through the convention by being transformed into a convention of “humanitarian, species, and nature” protection and dignity. I suggest CITES could be an instrument funneling economic resources from rich countries in the North to countries in the South, where their state budgets partly rely on wildlife trade. All rich countries could commit to transferring economic aid to the convention’s funding, which a secretariat/board could distribute. More specifically, this could be done through evaluation of applications sent to the secretariat that document local, regional, and national projects that aim to protect species from exploitation and to genuinely help endangered species and species not yet endangered to thrive, while providing economic resources to people working to achieve this goal. There are for example many projects ran by NGOs worldwide aiming to establish alternatives to wildlife trade for local communities.^9^ NGOs could apply to the convention for economic support to run these projects. CITES could be renamed *The Convention for the conservation of wild fauna and flora*, rather than being a trade convention.

In addition, aid from rich countries in the North to countries in the South that are rich in biodiversity but poorer in economic resources could be conditioned on the ways in which they succeed in protecting the natural environment and its habitants, which is a system already in place when it comes to the protection of rainforest. For example, Norway and Germany contribute significantly to the protection of rainforest in places such as Brazil, Colombia, and Ecuador through REDD +^10^; the amount of economic resources that are allocated from Norway to these countries depends on how much rainforest is spared from logging and the emissions that are reduced (Hermansen & Kasa, 2014; Sollund et al., 2019).

## Conclusion

This article has demonstrated how the partial legalization of the reptile trade in Norway has weakened the enforcement of laws against the illegal reptile trade. The partial legalization puts more animals at risk because the threshold for acquiring reptiles has been significantly lowered and the accessibility of the animals has increased through now legal internet marketplaces. The rules that have been set for this legal reptile trade are insufficient and easily circumvented; the risk of apprehension by law enforcement is minimal for the offender. From the animal victims’ perspective, however, when the law is enforced, the consequences are fatal because animals are consistently killed by the authorities. This is a paradoxical way of enforcing CITES (Sollund, 2019, 2021).

The harms caused by the wildlife trade and the lack of enforcement of CITES demand a remodeling of the convention. In the current nature crisis, new relations between humans and the non-human world are urgent and an important step would be to acknowledge wildlife crime as eco-violence and ecocide (Stoett & Omrow, 2020). Harms to nonhuman animals must be punished in proportion to the harms committed. This would imply that such crimes should not be addressed by means of administrative sanctions, with fines, but be taken to court where they should be punished as serious, environmental crimes, animal abuse, and theriocides (Sollund, 2013). This would treat animals who are trafficking victims as *victims*, rather than as property that can be instrumentally traded, killed, and discarded for individual deterrence and punishment.

Rather than relying only on punishment, strong economic incentives should be used to support people who protect species from wildlife trade and the threat of abuse and extinction. CITES could be remodeled to administer such economic support.

## Notes

1. “Animal” is a misleading and anthropocentric word because it categorizes all nonhuman animals in one category, despite the evident difference between for example, an elephant, a mouse, and a crab. It also implies that there is a contradiction between the human animal and other animals signifying that humans are not animals. For simplicity and with this reservation, I retain the word, “animal,” in this article because I have not yet come up with a short and good alternative. “Wildlife,” usually meant to include both plant and animal life, is another anthropocentric word in that it alienates free born animals and treats them as dangerous, primitive and unpredictable, particularly large carnivores. With regret and for simplicity, I retain this term in this article, but only to represent free born animals or animals who are bred but not adapted to a life in captivity under human control. Finally, “pet” is problematic insofar as it suggests that the purpose of an animal is to be company for and to be petted by a human. The implication, therefore, is that the animals serve no purpose other than their instrumental function for humans. “Pet” is also very often misleading because many animals who are sold a “pets” cannot be handled by humans, such as many reptiles. In previous work (e.g., Sollund, 2011), I used the word, “compan” as an abbreviation for companion animal. In the context of reptiles, I do not find this concept very accurate or helpful, so I employ “pet” to refer to animals who are made part of private households, whether or not this implies a social and/or physical relationship based on touch. Occasionally, I use “household animals” to describe the same beings. Many household animals are victims of violence and oppression, which makes the concept of a “pet” even more misleading.

2. Another declaration of animal rights extends further than the UNESCO declaration. The organization, *Our planet. Theirs too*, have formulated basic rights for animals that essentially prohibits any exploitation of animals and obliges humans to prosecute any harm committed against them (Our planet. Theirs too, 2011).

3. For the CRIMEANTHROP project, funded by the Norwegian Research Council, project number 289285, I have conducted 17 qualitative interviews with border veterinarians, customs inspectors, police, and advisors in the Norwegian Environment Agency. These data complement the approximately 800 penal case files and customs seizure reports, the analyzes of which are work in progress.

4. It needs mentioning though, that a revision of the CITES regulation in Norway in 2021 implied that: “Before living specimens of species included in Annex 1, List A, are destroyed, the Agency shall consider the possibility of relocating such specimens.” Regulation on import, export, domestic possession etc. of endangered species of wild fauna and flora (CITES Regulation)—Lovdata, Section 29. It is an empirical question whether this will have any effect for CITES I (list A) species. Appendix II species will likely still be killed.

5. Convention on the conservation of European wildlife and natural habitats (Bern Convention) (coe.int).

6. Økokrim in English—Økokrim (okokrim.no).

7. Lov om dyrevelferd—Lovdata.

8. “Sustainable development” was defined in the World Commission on Environment and Development’s 1987 Brundtland report “Our Common Future” as “development that meets the needs of the present without compromising the ability of future generations to meet their own needs” (see, e.g., Glossary of summaries—EUR-Lex [europa.eu]).

9. One such project is Entropika in Colombia that organizes projects for the local population in Leticia, providing them with income sources that are viable alternatives to wildlife trafficking. Entropika | Amazonas | Colombia. WWF is another important NGO in the field of wildlife conservation. See for example, wwf_doubling_tigers_annual_report___web_version.pdf (panda.org).

10. NICFI—Norway’s International Climate and Forest Initiative (NICFI).
